# Hepatorenal protective activity of Artemisia against diclofenac toxicity in male rats

**DOI:** 10.11604/pamj.2022.43.192.36160

**Published:** 2022-12-14

**Authors:** Shatha Hussein Kadhim, Amal Umran Mosa, Moayad Mijbil Ubaid

**Affiliations:** 1 Department of Pharmacology and Toxicology, College of Pharmacy, University of Kerbala, Karbala, Iraq,; 2 Department of Sciences, College of Basic Education, University of Sumer, Rifai, Iraq

**Keywords:** Artemisia, diclofenac, kidney criteria, liver enzymes

## Abstract

**Introduction:**

Artemisia is one of the important alternative treatments for many diseases, as well as the prevention of the effect of oxidizing substances that cause damage to the various organs of the body, including the liver and kidneys. The kidney and the liver are considered the body's most critical organs, and their functions in storage, metabolism, detoxification and elimination of medications, and their metabolic products make them target structures for “drug-induced” harm. The goal of this investigation was to see if Artemisia extract might protect hepatic and renal tissues from diclofenac-induced damage.

**Methods:**

a total of 40 adult Wistar rats were separated equally into four groups randomly. The rats of the control group got only distilled water orally without medicine or therapy, while those in the second group administrated 100mg/kg/day of Artemisia orally for one month. The third group received 10mg/kg/day of Diclofenac (DF) orally. The fourth group received 10mg/kg/day of DF and 100mg/kg day of Artemisia orally. After one month, kidney parameters (albumin, creatinine, and urea) and liver parameters (aspartate aminotransferase (AST), alanine aminotransferase (ALT), and alkaline phosphatase (ALP)) were measured.

**Results:**

the results revealed increasing in the kidney (albumin, creatinine, and urea) parameters and liver parameters (AST, ALT, and ALP) in the group treated with diclofenac compared to the control group while they decreased significantly (p≤0.05) in diclofenac + Artemisia group comparing to diclofenac group.

**Conclusion:**

we conclude from these results that Artemisia may have a role in reducing the toxic effect of diclofenac on kidney and liver by decreasing the liver enzymes and kidney criteria in the blood. The aim of the present study is to evaluate the role of Artemisia to reduce the toxic effect of diclofenac on liver and kidney.

## Introduction

Because of the abundance of active principles provided by nature during millions of years of evolution, medical valuable plants, serve a substantial role in modernistic medicine. Plant compounds, also known as phytochemicals, have a wide range of physiologically active, beneficial effects and help plants protect themselves against insects, bacteria, viruses, and other predators. To achieve the intended pharmacological effect, these phytochemicals operate on many pathways at the same time, either alone or in combination. Many medical valuable plants or herbs are venerable by traditional medical traditions (“Chinese medicine, Ayurveda, Native Americans, and so on”) because of their therapeutic abilities, and plants account for more than 40% of modern pharmaceuticals [1-3]. Modern medical studies are heavily focused on developing new anti-infectious drugs for the treatment of a variety of viral diseases like flu, hepatitis, herpes simplex virus (HSV), coxsackievirus, and HIV [4-7]. The first trial to use Artemisia extract as a drug is in malaria patients in August 1972. Following the conclusion of the trial, artemisinin is extracted and identified as the active ingredient in the extract of Artemisia. Recently, several of derivatives and man-made compounds with a structure similar to artemisinin have been made. Many cell lines of cancer, including leukemia, colon cancer, renal cell carcinoma, and breast cancer cells have been found to stop growing when Artemisia species are administrated [8-10]. The phytochemical analyses of various extracts of Artemisia revealed that they all included flavonoids, coumarins, cardiac glycosides, tannins, and anthocyanins. These phytochemicals and their derivative products slow down growth by, among other things, stopping angiogenesis, causing apoptosis or stopping the cell cycle, and affecting cell migration [11-13]. Annua's artesunate and piperaquine, as well as Judaica´s piperitone and trans-ethyl cinnamate, have antiviral and anti-inflammatory properties [14-17]. The World Health Organization (WHO) currently recommends a mixture of artemisinin and its derivative products (ARTs) for the medication of malaria [18].

Diclofenac (DF) is one of the drugs that belong to the family of nonsteroidal anti-inflammatory drugs (NSAIDs), and it is used to decrease pain, fever, and inflammation [19]. Diclofenac is believed to be one of the substances that causes liver and kidney cell harm, despite its therapeutic properties. The toxic effect of DF and its derivative (4,5-hydroxydiclofenac) have been connected with mitochondrion damage and deterioration of immune-mediated defense mechanisms [20]. In the kidney and liver cells, evidence also demonstrated that DF causes cell necrosis, which is associated with the generation of reactive oxygen species (ROS) and the suppression of enzymatic and non-enzymatic antioxidant activity. As a result, any medicinal reagent with antioxidant activity may reduce the cellular damage produced by ROS and can be upgraded to be a medicated attitude to prevent the toxic consequences of DF [21]. Diclofenac´s toxicity has been proven in numerous research [22,23]. The present study aims to evaluate the protective effect of Artemisia against diclofenac toxicity on the liver and the kidney.

## Methods

**Study size and design:** forty male Wistar albino rats, the weight ranged (230-250 g) and the age ranged (2-3 months), were used. The rats were separated into four groups in plastic cages, fed a regular food, and given tap water and exposed to 12-hour light-dark cycle for 28 days (during the experimental period). Each separated group comprising 10 animals: 1) Control group: the rats administrated only distilled water, no diclofenac nor Artemisia, for one month; 2) Artemisia group: from the first to the last day of the experiment, rats were given 100 mg/kg Artemisia daily orally through needle gavage; 3) diclofenac group: rats were given 10 mg/kg diclofenac orally every day from the first to the last day of the experiment; 4) Artemisia + diclofenac group: a dosage of 100 mg/kg of Artemisia and 10 mg/kg of diclofenac was given orally to rats via gavage every day from the first to the last day of the trial.

**Setting:** the current study was carried out in the animal house of College of Pharmacy, University of Kerbala, Karbala City which lies in the middle of Iraq for the period from January 5^th^, to March 3^rd^, 2022. The animal house is equipped with all the necessary means to preserve the experimental animals and unify the external conditions such as cages of the same sizes, heating and cooling devices, air vacuums and thermometers, in addition to the necessary tools used for feeding and drinking water. The animal house is also managed by a number of skilled technicians who have experience in dealing with laboratory animals.

**Variables:** the current study was experimental and depended on laboratory rats. They were randomly separated into four groups, control healthy rats, rats administrated diclofenac, rats administrated Artemisia, and rats administrated both Artemisia and diclofenac. All lab animals were exposed to the same conditions such as temperature, feeding, and light-dark period.

**Drug dose preparation:** dose of diclofenac, 10mg/kg.bw [24], was prepared using diclofenac tablets (50 mg) from the company (Gulf Pharmaceutical Industries). Tablets were grinded and dissolved in drinking water. Every tablet was dissolved in 5 ml of drinking water to get the stock solution of the dosage, 10mg/ml of diclofenac, which was standard for 1kg of body weight. To find the dosage for each rat according to its weight the following equation was practiced:


$$Ml\text{ of stock solution for each rat = }\frac{Weight\text{ of rat (mg)}}{1000}$$

As an example, the dosage for 240 gm rat = 240/1000 = 0.24ml of stock solution which equal to 10mg/kg.bw.

**Plant extract preparation:** the air-dried herb of *Artemisia annua* powder (100 g) was combined with one liter of heated distilled water, preserved at 60°C for 15 minutes, and afterward the mixture was filtered three times by filter paper. The resultant solution was left to dry at 37°C until it left behind a 62 percent w/w concentrated residue. The stock solution was preserved at -20°C until it was used, and normal saline was used to make up the diluted concentration [25].

**Serum collection:** at the end of the experiment, two ml of blood was taken from each rat through a syringe, and then placed in a test tube and left for half an hour to clot, then the test tubes were transferred to the centrifuge, where the tubes were centrifuged at a speed of 4500 rpm for 10 minutes to separate serum from coagulant blood, which was subsequently frozen until biochemical examinations were done.

**Laboratory measurements:** biochemical tests for kidney function including aspartate aminotransferase (AST), alanine aminotransferase (ALT), and alkaline phosphatase (ALP) in addition to liver function including albumin, creatinine, and urea were done for all groups of the study (control, Artemisia, diclofenac, and dicfofenac + Artemisia) groups. A Dri-Chem-NX500 auto-analyzer was used to measure biochemical analyses in blood samples (Fujifilm Corporations, Japan). Dry-Chem analyzer was a dry chemistry analyzer that can carry out a variety of clinical chemistry test parameters. It includes an integrated auto-pipetting system, required no calibration or water, made setup and maintenance simple.

**Bias:** the current study was an experimental study conducted on 40 male rats under controlled conditions in the laboratory, where the rats were selected of close ages and weights and from the same breed, and randomly divided into four groups and exposed to the same environmental conditions such as temperature, lighting period, and nutrition throughout the experiment period.

**Data analysis:** statistical Package for the Social Sciences (SPSS) for windows operating system was used for statistical analysis. All data were reported as a Mean ± SE. The statistical analysis by using a “One-way analysis of variances” was done to examine the statistical significance of differences between the control and other groups (ANOVA, p≤0.05).

**Ethics issues:** the experiment of the present study was done according to instructions of animal ethics committee in Pharmacy Faculty, University of Kerbala, where the study was done, clearance number (Ph3423).

## Results

**Participant:** the current experimental study was conducted on 40 male rats under controlled conditions in the laboratory. The rats were randomly assigned into 4 groups, each group containing 10 rats. All rats were close in age and weight and of the same breed. At the end of the experiment, the kidney functions represented by creatinine, albumin, and urea criteria, in addition to the liver functions represented by alanine and catalase enzymes, were measured and the following results were revealed.

**Kidney function:** Table 1 revealed a significant increase (p≤0.05) in albumin, creatinine, and urea in the diclofenac group (4.84±0.59, 0.39±0.02, 63.00±4.46 respectively) compared to the control group (3.03±0.02, 0.26±0.01 and 39.50±4.31 respectively). While it decreased significantly (p≤0.05) (3.46±0.27, 0.28±0.02 and 48.00±6.11 respectively) in diclofenac + Artemisia group comparing to diclofenac group (Table 1).

**Table 1:** the effect of Artemisia and diclofenac on the level of albumin, creatinine, and urea in the serum of the male rats

Groups	Albumin (mg/dl) mean ± SE	Creatinine (mg/dl) mean ± SE	Urea (mg/dl) mean ± SE
Control	3.03 ± 0.2^c^	0.26 ± 0.01^b^	39.50 ± 4.31^b^
Artemisia	2.84 ± 0.15^c^	0.22 ± 0.01^b^	33.83 ± 2.35^c^
Diclofenac	4.84 ± 0.59^a^	0.39 ± 0.02^a^	63.00 ± 4.46^a^
Diclofenac + Artemisia	3.46 ± 0.27^b^	0.28 ± 0.02^b^	48.00 ± 6.11^b^

a, b, c : significant difference p≤ 0.05

**Liver function:** Table 2 revealed a significant elevation (p≤0.05) in AST, ALP, and ALT in the diclofenac group (90.50±8.09, 280.50±17.16 and 65.83±6.30 respectively) comparing to control group (61.50±7.06, 164.33±13.56 and 42±3.46 respectively). While it decreased significantly (p≤0.05), (66.00±5.13, 140.50±16.18 and 43.83±5.96 respectively) in diclofenac + Artemisia group comparing to diclofenac group.

**Table 2:** the effect of Artemisia and diclofenac on AST, ALP and, ALT in the serum of the male rats

Groups	AST(U/L) mean ± SE	ALP(U/L) mean ± SE	ALT(U/L) mean ± SE
Control	61.50 ±7.06^b^	164.33 ± 13.56^b^	42 ± 3.46^b^
Artemisia	58.33 ± 4.27^b^	142.16 ± 15.54^b^	40.16 ± 2.05^b^
Diclofenac	90.50 ± 8.09^a^	280.50 ± 17.16^a^	65.83 ± 6.30^a^
Diclofenac + Artemisia	66.00 ± 5.13^b^	140.50 ± 16.18^b^	43.83 ± 5.96^b^

a, b, c : significant difference p≤ 0.05; AST: aspartate aminotransferase; ALP: alkaline phosphatase; ALT: alanine aminotransferase

## Discussion

The toxicity of the liver and kidney, which is based on distinct pharmacological mechanisms, has a wide range of consequences resulting from cell destruction and causing acute or chronic functional abnormalities in the respective organs [26]. So, there is a significant increase in (ALT, AST, and ALP) values, which could be due to hepatocellular damage caused by DF toxicity, which caused these enzymes to diffuse from damaged liver cells to the bloodstream. The Artemisia plant has the unusual characteristic of adjusting reactive oxygen species (ROS). It shows a significant antioxidant and radical scavenging action against OH ion and H_2_O_2_, and it provides excellent protection by enhancing the antioxidant defense system and decreasing the generation of reactive oxygen species (ROS) [6,27]. Artemisinins (ARTs) function by binding to Fe^+2^ (e.g. heme) and producing reactive oxygen species (ROS), which can be cytostatic or cytotoxic. Reactive oxygen species can cause cellular injury through the peroxidation of membrane phospholipids, the activating of pro-apoptotic mechanisms, and the instability of genomic and mitochondrial deoxy-ribonucleic acid (DNA) [28]. Artemisinin, artesunate, dihydroartemisin, scoparone, capillarisin, eupatilin, and scopoletin artemisolide are some of the anti-inflammatory sesquiterpenes extracted from Artemisia and its derivatives. In animal models, ARTs are effective in the medication of inflammatory diseases such as systemic lupus erythematosus, rheumatoid arthritis, multiple sclerosis, and allergy disorders.

The Artemisia plant has the unusual characteristics of modulating ROS. Sometimes in cases, they have a strong effect on hydroxyl ions and hydrogen peroxide as antioxidants and radical scavengers. They also may offer good protection by making antioxidants work better and reducing the production of ROS [6,27]. Artemisinins work by binding to fe^+2^(like heme) and making ROS, which can either stop the growth of cells or kill them. ROS can damage cells by making it easier for membrane phospholipids to oxidize, by turning on pro-apoptotic pathways, and by making genomic and mitochondrial DNA less stable [28]. Some of the anti-inflammatory sesquiterpenes that can be taken from Artemisia and its derivatives are “artemisinin, artesunate, dihydroartemisin, artemisolide, eupatilin, scoparone, capillarisin, and scopoletin”. In animal models, ARTs are effective in the healing of many inflammations like “rheumatoid arthritis, systemic lupus erythematosus, multiple sclerosis, and allergy disorders” [29]. Our findings suggest that Artemisia may have a therapeutic effect on liver and kidney parameters by lowering high levels caused by diclofenac toxicity, which is consistent with [2,3].

Parasuraman *et al*. [2] and Barkat *et al*. [3] discovered that the Artemisia genus contains a wealth of bioactive components that have anti-inflammatory properties. The antioxidant activity of different Artemisia species components is studied, particularly the “n-butanol” extract, which lead to a significant elevation of cytosolic superoxide dismutase (SOD) and catalase in rat liver, as measured by enzyme-linked immunosorbent assay (ELISA) [15,30]. Kumar *et al*. [31] discovered that the antioxidant characteristics of Artemisia annua of aerial parts are equivalent to 18% of the reference component (alpha-tocopherol). On the other hand, diclofenac (DF) is considered one of the drugs that cause liver and kidney cell harm. Several studies and research have linked the toxic effects of DF´s and its metabolites (“4’, 5-hydroxydiclofenac”) to the damage of mitochondria and the impairment of immune-mediated protective systems [20,26,32].

In addition, the actions of enzymatic and non-enzymatic antioxidants in liver and kidney cells are inhibited. As a result, any medicinal reagent with antioxidant activity can reduce cellular damage produced by ROS and can be upgraded to be a medicated attitude to avoid DF harmful effects [21]. A great deal of evidence suggests that oxidative stress is to blame for DF toxicity and harmful consequences [33].

**Limitations:** in the current study, we used only adult and healthy male rats, while female rats were excluded also, low active rats were excluded.

## Conclusion

We can conclude from this study that Artemisia may have hepatorenal protective and preventive effects against diclofenac-induced liver and kidney damage. Finally, the results of this investigation showed that Artemisia may have preventive effect against liver and kidney cell damage caused by a variety of drugs, including nonsteroidal anti-inflammatory drugs (NSAIDs). However, further research is needed in humans to validate the efficacy, therapeutic effects, adverse effects, and exact dosing.

### What is known about this topic

- Herbal medication is a good choice for the treatment of different disorders caused by chemical drugs.

### What this study adds

- Artemisia has a surprising effect in reducing the toxic effect of nonsteroidal anti-inflammatory drug on liver and kidney.
